# Identifying the criteria for community-centred Life Cycle Sustainability Assessment of estate regeneration schemes

**DOI:** 10.1016/j.heliyon.2024.e31115

**Published:** 2024-05-13

**Authors:** Sahar Nava, Zaid Chalabi, Sarah Bell, Pablo Sendra, Esfand Burman

**Affiliations:** a University College London, United Kingdom; b University of Melbourne, Australia

**Keywords:** Life cycle sustainability assessment, Sustainability indicators, Impact criteria, Social housing regeneration, Estate regeneration, Participatory research

## Abstract

Identifying the overall environmental, and socioeconomic impacts of different estate regeneration scenarios can contribute to the overall sustainability of such schemes. Life Cycle Sustainability Assessment (LCSA) is an appropriate tool for assessing holistic sustainability. To achieve resilient societies, the interests of communities should be considered in decision making. This paper proposes a method for incorporating community needs in identifying sustainability metrics for the sustainability assessment of estate regeneration schemes. A literature review in the field of sustainability assessment of buildings is conducted followed by a mixed methods empirical research. Collection of data has been through surveys, an interview, and an evaluation questionnaire. Data has been analysed through statistical and thematic analysis and triangulation of the results. The findings have consistently yielded the limitations of the scope of the current sustainability assessment methodologies, especially for lack of attention to societal impacts of regeneration. The results have justified the need for this research to employ participatory approaches for identifying a relevant set of sustainability indicators and criteria for assessing the lifetime impacts of estate regeneration schemes. Issues related to community involvement in decision making, maintenance and management, community facilities, refurbishment, and disruption have been identified as the stakeholders’ top priorities. Mental Health and Socioeconomic Values have been introduced as new criteria. The findings confirm the need for an in-depth approach towards identifying the regeneration priorities of the communities for the scope of LCSA studies. The identified list of criteria can apply to other studies of this context for an equitable approach for selecting the indicators across different criteria and for communicating the LCSA results with different stakeholders.

## Acronyms

BRE: Building Research Establishment
BREEAM: Building Research Establishment Environmental Assessment Method
BS: British Standards
BSI: British Standards Institute
BSAS: Building Sustainability Assessment Systems
GHG: Green House Gas
GLA: Greater London Authority
HQM: Home Quality Mark
ISO: International Organization for Standardization
LCA: Life Cycle Assessment
LCSA: Life Cycle Sustainability Assessment
RIBA: Royal Institute of British Architects
TA: Thematic Analysis
TBL: Triple Bottom Line

## 1 Introduction

To achieve sustainability in a society, a balance between different environmental and socioeconomic impacts of different building and infrastructure projects is required [1]. Since the UK has pledged to reduce its net Greenhouse Gas (GHG) emissions by 100 % by 2050 compared to 1990 levels [2], a lot of attention has been brought to the assessment of carbon emissions of buildings over their lifecycle. The methodological and practical limitations of a carbon emission-centred and environmental-focused approach can lead to discrepancies in the assessment results [3]. Kloepffer and Renner [4] introduced Life Cycle Sustainability Assessment (LCSA) in 2008 [5] as a methodology aimed at evaluating the overall sustainability of products and systems [6]. LCSA has proven valuable in aiding decision-making for building schemes, as it effectively assesses the environmental, social, and economic impacts associated with such projects [6,7].

There is still a lack of standardisation of the indicators and criteria used in the assessment methodologies. In addition, most existing literature does not include stakeholders in the process of identifying the sustainability indicators [1,8]. Not only there is ample evidence of the positive impacts of stakeholder involvement in conducting sustainability assessment, but full stakeholder engagement in development of proposals is also mandated in many countries. In the UK, the legislation is under the Sustainable Communities Act 2007 [9]. The 2018 requirement by the Mayor of London obliges resident ballot as a condition for estate landlords applying for funding managed by 10.13039/100013998Greater London Authority(GLA) for building affordable housing [10]. The requirement for ballots has resulted in more attention being directed towards participatory options appraisal of regeneration schemes in London. However, there is little scrutiny in identifying the community's priorities in relation to estate regeneration schemes. Identifying these priorities can assist in establishing the sustainability indicators for the assessment of regeneration scenarios. Sala et al. [11]*,* Zamangi et al. [12], and Souza et al. [13] have raised the importance of stakeholder involvement in the selection of sustainability indicators as one of the main gaps in conducting sustainability assessments. Laurin et al. [14] note the importance of building users and designers in identifying which categories to include in the scope. To achieve holistic and viable sustainable outcomes, the stakeholders should be engaged and their expectations and perspectives should be considered especially in the context of sustainable retrofit projects [15].

This paper investigates one element of a larger study in developing a participatory sustainability assessment framework for the appraisal of estate regeneration schemes. The study aims to identify a global list of criteria for a community-centred LCSA framework of estate regeneration schemes. We have applied some of the existing approaches to criteria selection and eliciting the priorities of the community on a mixed methods case study.

The research aim is to identify a set of criteria for community-centred LCSA of estate regeneration schemes. This is achieved through the below objectives;

1. Identifying gaps in the classification of sustainability criteria,
2. Identifying the most common and relevant sustainability indicators and criteria used in research and practice,
3. Identifying the community priorities in relation to regeneration schemes.

Objectives 1 and 2 were explored via a literature review including the review of standards, legislation, and other relevant documents. The collection of primary data to respond to objective 3 has been explored in a single-case mixed methods case study consisting of two surveys, an interview, and an evaluation questionnaire.

**Fig. 1:**
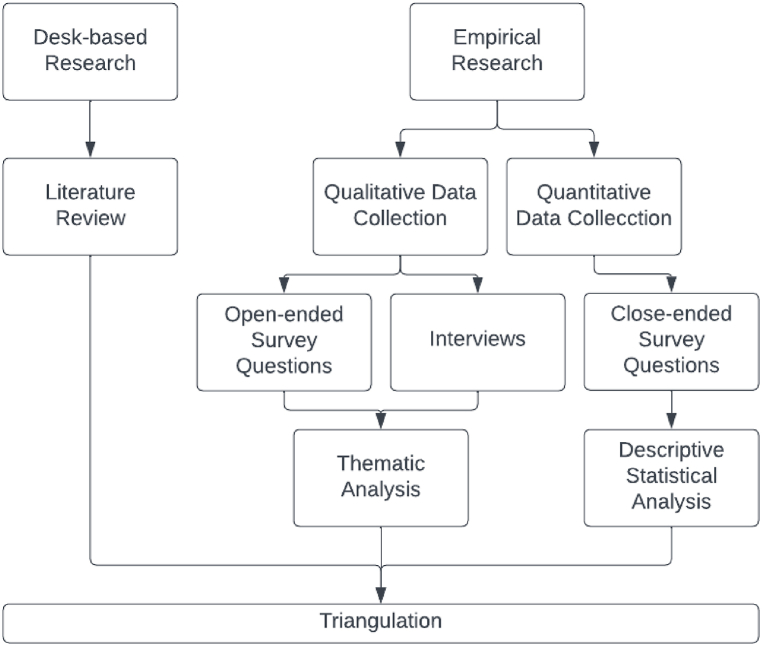
Research flow diagram.

Fig. 1 illustrates the flow of this research.

## 2 Literature review

A review was conducted to identify the scope of an LCSA framework in the context of the building industry with more focus on the UK context. The review recognises global indicators and categorisation of those indicators for different LCSA and sustainability assessment methods. It also reveals the gaps in the field for identifying the indicators and categorisation of criteria, and highlights the importance of this review in introducing a global set of criteria for a more transparent participatory approach and communication of the results.

**Fig. 2:**
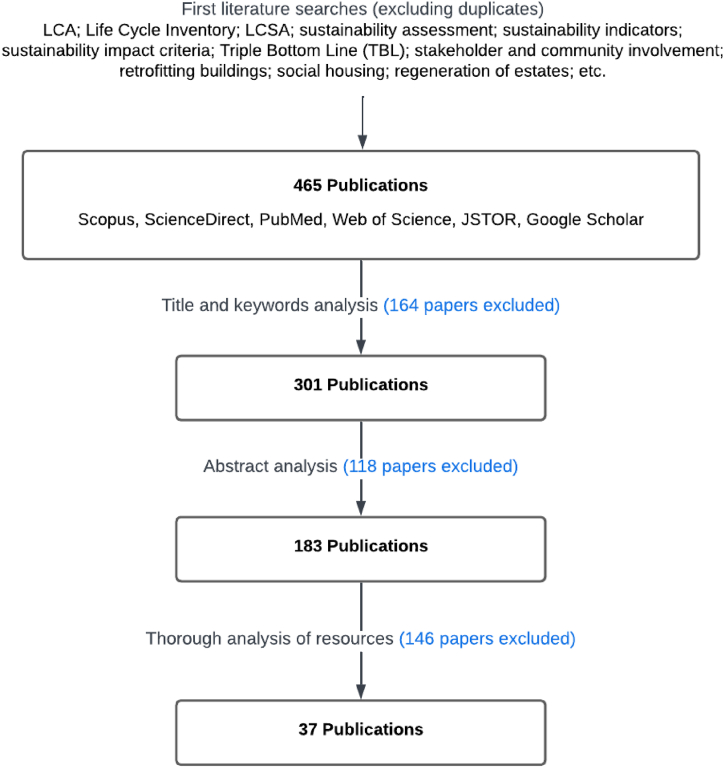
Summary of the procedure for selection of literature sources for scoping review. Own elaboration from PRISMA-ScR guidelines [16].

To conduct this review, PRISMA-ScR guidelines and methods have been employed [[16], [17], [18]]. The search has been conducted on recent English-written publications, mostly within the last 10 years, except for recognised sources of earlier literature. In selecting the publication, it has been considered for the literature to have been either peer-reviewed or well-recognised in the industry. Scopus, ScienceDirect, PubMed, Web of Science, JSTOR, and Google Scholar were the main sources of evidence. Each publication was independently reviewed to make sure that it responded to the research question and its objectives. The following search terms were used to retrieve the articles from the databases: Life Cycle Assessment (LCA); Life Cycle Analysis; Life Cycle Inventory; Life Cycle Sustainability Assessment (LCSA); sustainability assessment; sustainability indicators; sustainability impact criteria; Triple Bottom Line (TBL) approach; stakeholder and community involvement in decision-making; retrofitting buildings; social housing; and regeneration of estates. A coding system was applied in parallel with Mendeley referencing manager [19] for categorising and synthesising data. Four-hundred and sixty-five items were initially evidenced, each of which was screened to make sure the dates, fields, and research were relevant. Fig. 2 presents a summary of the procedure for the selection of literature for the scoping review. After analysing the resources, the final list for identifying the sustainability indicators and criteria included thirty-seven publications presented in Table 1.

**Table 1:** List of the final *thirty-seven* references for the scoping review.

Literature Type	Literature References
References from Standardisation, Sustainability Assessment, and Certification Scheme Documents	[[20], [21], [22], [23], [24], [25], [26], [27], [28], [29], [30], [31]]
References from Academic and Journal Publications	[[1], [5], [8], [15], [32], [33], [34], [35], [36], [37], [38], [39], [40], [41], [42], [43], [44], [45], [46], [47], [48], [49], [50], [51], [52]]

The review found inconsistency and incomprehensiveness of criteria classification, lack of engagement with the communities in identifying their priorities for the selection of indicators and criteria, and lack of contextual considerations for sustainability assessment of estates as the main gaps in identifying the LCSA scope.

### 2.1 Holistic sustainability assessment

The term LCA mainly dates back to the 1970s and 1980s [6]. The methodology used for assessing the overall ‘sustainability’ of [building] products and systems, consisting of the assessment of environmental, social, and economic impacts of different scenarios is referred to as LCSA [6](7). The impact criteria in different studies vary from reductionist scopes, categorising environmental; social and economic impacts, to more ‘holistic’ approaches in which different quantitative and qualitative dimensions of sustainability are integrated [40].

In recent years, LCSA has become a more promoted tool for specialists to employ in different phases of their design [53]. However, due to the specialist nature of the studies and lack of involvement with the communities, the priorities of different stakeholders are not considered in the decision-making over the scope of the studies.

### 2.2 Sustainability indicators

**Fig. 3:**
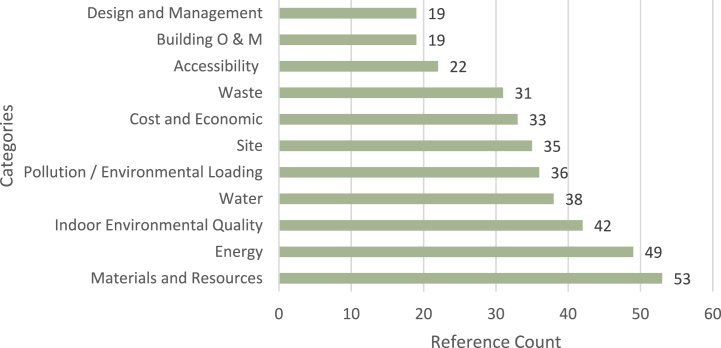
– Authors' reproduction of the top identified categories by Lazar and Chithra [48].

A comprehensive review of seventy-eight publications in relation to Building Sustainability Assessment Systems (BSAS) by Lazar and Chithra [48] has identified that fifty-eight out of the seventy-eight publications have considered categories as a level in their hierarchy tree. Out of the identified forty-five categories weighted based on the number of references, Materials and Resources, Energy, and Indoor Environmental Quality have the highest ranks followed by Water and Pollution [48]. Fig. 3 illustrates the authors’ re-production of the highest ranked results of Lazar and Chithra [48] review. Similarly, Amini Toosi et al. [53] have noted the higher rank of environmental and economic aspects compared to social dimensions.

**Fig. 4:**
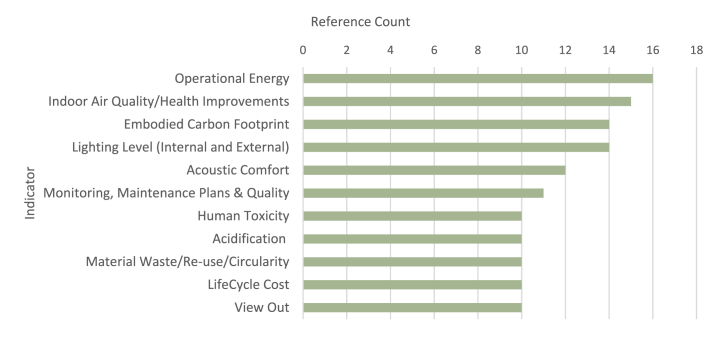
A summary of the top 10 identified impact categories and indicators through the literature review.

Our extensive review of thirty-seven publications in relation to Building LCSA and sustainability assessment schemes (consisting of the identified schemes and standards) has been conducted to extract a prolonged list of sustainability indicators. Fig. 4 illustrates the bar chart of our top identified indicators weighted based on their number of references across the dataset. There has been a mixed-use of mid-point and end-point [54],^1^ impact criteria across different literature in the identified list. In this paper, we refer to the mid-point criteria as sustainability indicators. Overall, it can be understood from this diagram that the most mentioned indicators relate to energy, health and well-being, and embodied carbon indicators. These results are aligned with the findings of Lazar and Chithra [48] and Amini Toosi et al. [53] on the energy-centric nature of the assessments and reiterate that social issues are amongst the least mentioned indicators.

### 2.3 Impact criteria

In addressing the sustainability indicators and their categorisation, more particularly in the context of housing estates, there has not been a consistent approach. Many studies divide sustainability criteria into broad categories of environmental, social, economic, and technical impacts for assessment [[55], [56], [57]]. A broad categorisation in classifying the impacts does not take into account the cross-criterion impacts [11,57] and can be prone to subjective interpretation for interrelated and interdependent categories. BS EN 15643:2021 [24] and BS ISO 21931–1:2022 [26] which are standard frameworks for assessment of buildings, engineering works, and construction works divide sustainability criteria into broad aspects of environmental; social; and economic impacts. These standards then provide a breakdown of these aspects into narrower mid-level categories, each of which includes a set of indicators. In this review, we have explored the categories proposed by different studies to compare the criteria for LCSA and sustainability assessment schemes.

Although not a lifecycle-based methodology, the Building Research Establishment Environmental Assessment Method (BREEAM) and Leadership in Energy and Environmental Design (LEED) are widely acknowledged as reputable sustainability certification schemes globally for the sustainability assessment of building projects. While primarily a voluntary scheme, many UK governmental organisations and public authorities now require a proposed building development to achieve a high BREEAM score [58]. The most relevant schemes in the UK in the context of housing estate regeneration projects depending on different scenarios of refurbishment, redevelopment, or a hybrid scenario, are Home Quality Mark (HQM) scheme [28]; BREEAM Community [23]; BREEAM Domestic Refurbishment [21], and BREEAM UK New Construction 2018 [20,59],^2^ and LEED V4.0 [27].

A comparison of the list of the criteria for each of these schemes with BS ISO 21931–1:2022 [26] has been presented in Table 2, illustrating how the criteria vary between different schemes.

**Table 2:** Comparison of the criteria of relevant sustainability assessment schemes.

BREEAM Domestic Refurbishment	BREEAM UK New Construction	BREEAM Community	LEED V4.0	HQM	BS ISO 21931–1:2022
Management	Management	Governance	Climate Change	Transport and Movement	Environmental Impacts
Health & Wellbeing	Health & Wellbeing	Social and Economic Wellbeing	Human Health	Outdoors	Environmental Aspects
Energy	Energy	Resources and Energy	Water Resources	Safety and Resilience	Stakeholder Aspects
Water	Transport	Landuse and Ecology	Biodiversity	Comfort	Accessibility
Materials	Water	Transport and Movement	Material Resources	Energy	Adaptability
Waste	Materials		Green Economy	Materials	Health and Wellbeing Characteristics
Pollution	Waste		Community Health	Space	Impacts on Neighbourhood
Innovation	Land Use and Ecology			Water	Safety and Security
	Pollution			Quality Assurance	Maintainability
	Innovation			Construction Impacts	Architectural Quality
				Customer Experience	Economic Issues
					Management Issues
					Additional Issues

The inconsistency in addressing the criteria for different assessment schemes is also reflected in addressing the sustainability indicators of those schemes. While British Standards Institute (BSI) [26] recommends a list of indicators for LCSA to be applied Europe-wide, the core indicators do not take into account the contextual considerations of different building types and locations, and there has not been a standardised scope for conducting the LCSA of regeneration schemes. Despite some of the schemes and guideline references of this review such as BREEAM and RIBA guidelines [60] being well-adopted to the UK legislation and practice, the references lack a number of important indicators mostly related to social and socioeconomic aspects. A common gap in the scope of most sustainability assessment methods is that the list of sustainability indicators may not necessarily be tailored to the context of different societies and cultures [8].

### 2.4 Gaps in literature

In exploring the core sustainability indicators and categories in LCSA and sustainability assessment frameworks, gaps in research, as well as practical limitations have been identified through this review as.

- incomprehensiveness of criteria classification,
- lack of community focus and engagement in identifying the sustainability indicators and criteria,
- lack of contextual considerations for sustainability criteria of estate regeneration

The empirical research of this paper intended to complement this review by engaging with the community of an estate and considering the priorities of the communities and residents of the study in the selection of assessment criteria. The results have identified a local set of community-based sustainability indicators and a global set of criteria for the scope of a community-centred LCSA for estate regeneration schemes.

## 3 Empirical research

As our review of the literature has revealed, the environmental impact categories in LCSA have been well-established. What lacks scrutiny in identifying a stakeholder-based scope for LCSA is the stakeholders’ perspectives on the environmental and socioeconomic impacts [13]. This study focused on including local knowledge from communities and exploring their perception of sustainability priorities concerning the regeneration of their estate.

We employed a mixed methods design which involved integrating and combining qualitative and quantitative data collection and analysis [61].

The single-case case study was on Alton Estate, which was an estate under threat of demolition in London. The council had approved a demolition and redevelopment masterplan for the regeneration of the estate in October2, 020.^3^ The main survey used for this study was conducted in collaboration between a team of researchers and the community of the estate [62]. The methods used in this study include surveys with open-ended and multiple-choice questions, a semi-structured interview, and a seven-point-scaling questionnaire held at a community workshop. Recruitment of the participants was made through Alton Action community group. Analysis of the survey questions in relation to regeneration priorities, and the interview results were used to answer one of the case study objectives of identifying the relevant indicators and criteria based on the community's priorities for estate regeneration. Table 3 illustrates an overview of the qualitative and quantitative approaches to data collection and analysis of this mixed methods study.

**Table 3:**
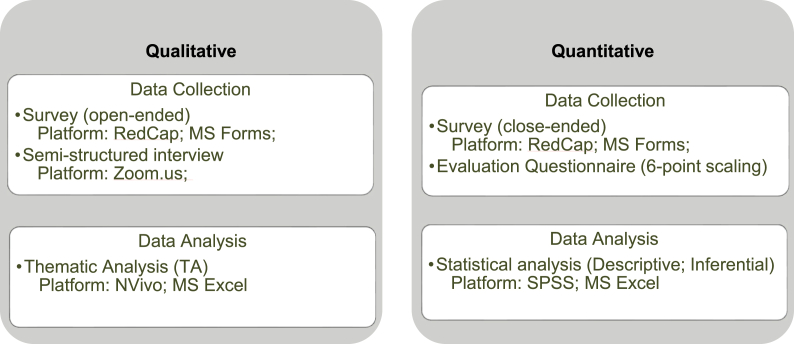
Data collection and analysis approaches of the case study.

Forty-seven participants took part in the main mixed-methods survey [62]. Six participants took part in the mostly qualitative second survey which was conducted through Microsoft Forms. The evaluation questionnaire had twenty-four participants. The main survey with forty-seven participants was used for quantitative and qualitative analysis. The second survey and the interview were analysed qualitatively. While there is no one formula for determining the sample size for mixed-methods research, the sample size depends on the specific research goals, objectives, and questions [63]. Creswell and Plano Clark [63] note the importance of collecting in-depth answers specifically for the qualitative part of the research, instead of increasing the number of participants. The mixed approaches of this study have complemented each other and have assisted in responding to the research aim and objectives of this study.

The main survey data was collected online using “*REDCap electronic data capture tools”* hosted at University College London.^4^,^5^ *“REDCap (Research Electronic Data Capture) is a secure, web-based application designed to* support *data capture for research studies, providing: 1) an intuitive interface for validated data entry; 2) audit trails for tracking data manipulation and export procedures; 3) automated export procedures for seamless data downloads to common statistical packages; and 4) procedures for importing data from external sources”*.^6^

The collected data from the close-ended survey questions of the main survey and the evaluation questionnaire were analysed through statistical analysis. The open-ended survey questions and the interview responses were analysed through Thematic Analysis. The findings of the qualitative and quantitative analyses were triangulated to complement the findings of the literature review and for discussion and conclusion.

### 3.1 Quantitative research

Questions relating to the participants' living conditions and their perception of the priorities of the estate and the buildings within the estate helped in identifying the community priorities as criteria for sustainability assessment. These questions explored issues such as the residents' satisfaction with their homes in terms of size, ventilation, the amount of daylight, and location, as well as the maintenance and management of the buildings and the estate, summer and winter temperatures at home and in the communal areas, living conditions, the participants' perception of their attachment to their homes, the impacts of the homes on participants’ health, and their preferences for relocation and its potential impacts.

The quantitative data from the survey has been explored using SPSS software [64] for descriptive and inferential statistical analysis. The measurement levels of the quantitative questions in both surveys were either of a nominal, ordinal, or interval scale. Descriptive statistics in terms of graphical bar charts, histograms, and scatter plots have been initially produced for the observational data. Crosstabulation analysis has been employed as a recommended approach for the analysis of categorical data using the chi-square and Cramer's V tests to examine the likelihood of associations between different variables [65,66].

### 3.2 Qualitative research

The qualitative research employed a mostly inductive approach in gauging the perception of the participants on the regeneration of their estate. The qualitative approaches of this study consisted of open-ended survey questions and a semi-structured interview.

Analysis of the qualitative data was through reflexive TA to find the priorities of the community in order to reflect them in identifying the scope for LCSA of estate regeneration schemes. Relevant qualitative data from different approaches across all studies consisting of open-ended survey questions and the interview have been coded through an iterative process of coding using NVivo software [67] to uncover underlying themes [68]. The analytic patterns have been coded and developed based on Braun and Clarke's [69] guidance as one cohesive dataset across different collected data. The reflexive TA approach has been incorporated as a method recommended for the analysis of participatory approaches due to its accessibility [69]. Braun and Clarke's [69] guidelines have been incorporated concurrently to analyse the data in parallel to the iterative three-stage process of open coding, axial coding, and focused coding proposed by Bergin [68].

### 3.3 Evaluation questionnaire

Following the statistical and thematic analyses, a workshop was conducted to discuss the findings of this study with the community of the estate. At this workshop twenty-four participants from different stakeholder groups of the estate were present. Two of the researchers from the collaborative study [62] who were not available on the day of the workshop, separately took part in the workshop online. An evaluation questionnaire was conducted to ask to what extent the participants agreed with including the identified criteria for the assessment of the estates and to receive feedback for improving the workshop. Twenty-one stakeholders responded to the questionnaire, excluding the authors of this paper. Seven-point scaling was used to gauge the participants’ feedback.

## 4 Findings

### 4.1 Results of literature review

To find a coherent list of criteria that is descriptive of the indicators and reflects the categories of different assessment methodologies, the identified impact categories from the literature review have been grouped into the criteria presented in Table 4.

**Table 4:** Authors’ combined list of criteria extracted from relevant sustainability assessment schemes.

	Criteria
1	Environmental Impacts & Strategies
2	Local Ecologic Impacts, & Strategies
3	Material Strategies and Circularity
4	Whole Life Cost
5	Health and Wellbeing
6	Accessibility
7	Safety and Security
8	Transport & Movement
9	Community Facilities and Amenities
10	Social Values
11	Management and Maintenance
12	Design Strategies and Innovation

Reviewing the relevant sustainability assessment schemes has revealed a combined set of 12 criteria being the most comprehensive categorisation for sustainability assessment of schemes.

As our extensive review of literature in the context of Building LCSA has revealed, similar to the findings of Lazar and Chithra [48], impact categories related to global warming are noticeably amongst the most studied categories. Since such indicators have been examined separately in some of the identified sustainability schemes, such as LEED V4.0 [27], and provided the recent attention to Net Zero trajectories [70], we have separated climate change from other environmental impacts and strategies. Table 5 presents our proposed list of criteria for LCSA scope from the review of literature.

**Table 5:** Authors list of criteria for LCSA extracted from the review of literature.

	Criteria
1	Climate Change
2	Environmental Impacts & Strategies
3	Local Ecologic Impacts, & Strategies
4	Material Strategies and Circularity
5	Whole Life Cost
6	Health and Wellbeing
7	Accessibility
8	Safety and Security
9	Transport & Movement
10	Community Facilities and Amenities
11	Social Values
12	Management & Maintenance
13	Design Strategies and Innovation

### 4.2 Statistical analysis of quantitative data

Statistical analysis of the quantitative survey questions has revealed some correlations between the participants' priorities and their preferred regeneration scenarios. Despite the participants' dissatisfaction with the management and maintenance of the buildings, the majority of the participants preferred a refurbishment scenario over demolition. The community's preference for refurbishment against redevelopment despite their dissatisfaction with certain aspects of the estate reveals that hidden criteria are impacting the community's priorities over their preferences for the regeneration plans. These results justify the need for qualitative research for an in-depth understanding of the community's priorities and identifying a scope for LCSA that reflects those priorities.

**Fig. 5:**
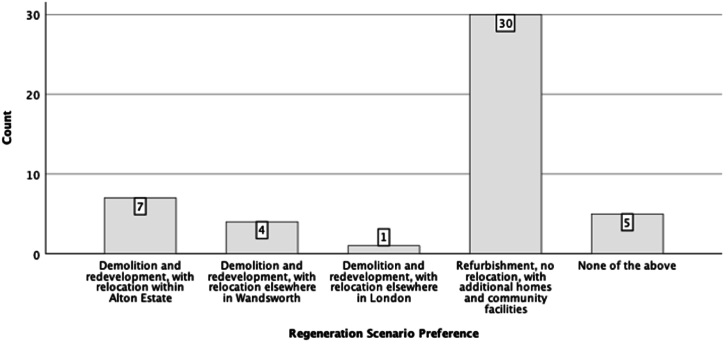
Histogram count of participants' preferred regeneration scenarios.

The survey included a question about the participants' preferred regeneration scenario. A histogram of the count of regeneration preference is presented in Fig. 5. The histogram highlights that the majority of the participants (63.8 % of the respondents) have selected refurbishment over other scenarios. The least favourite scenario is ‘demolition and redevelopment with relocation elsewhere in London’, with only one vote.

**Fig. 6:**
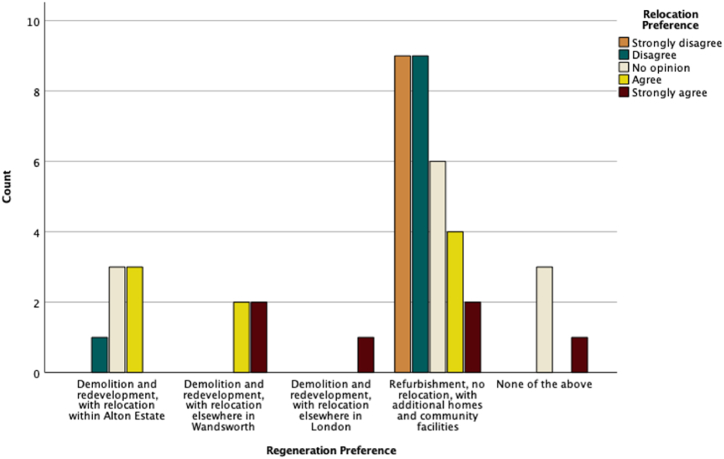
Graph showing Regeneration Preference against Relocation Preference.

**Fig. 7:**
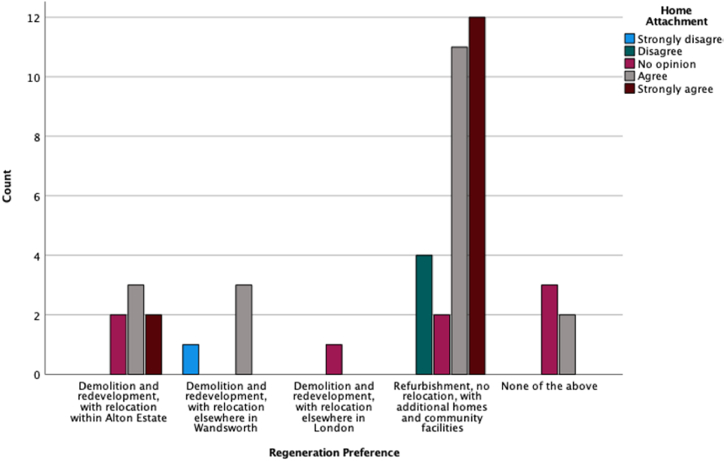
Graph representing Regeneration Preference against Home Attachment.

**Fig. 8:**
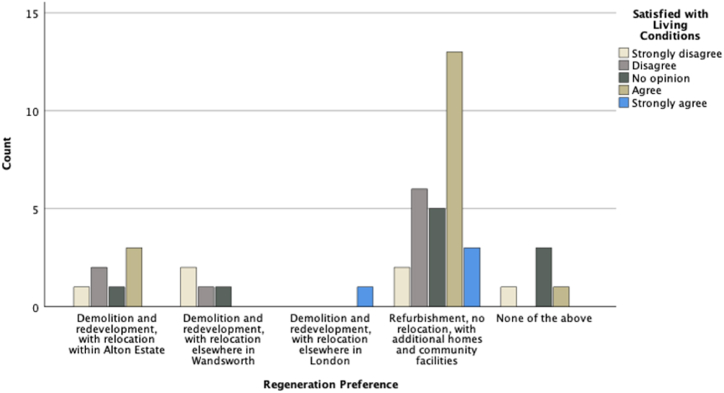
Graph representing regeneration preference against being satisfied with living condition.

**Fig. 9:**
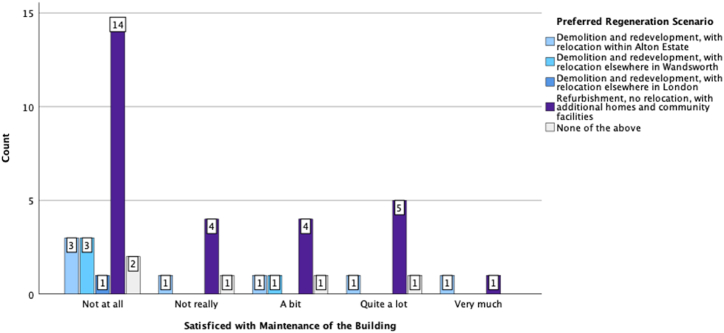
Grouped bar chart of regeneration preference compared to participants' satisfaction with the maintenance of the building.

**Fig. 10:**
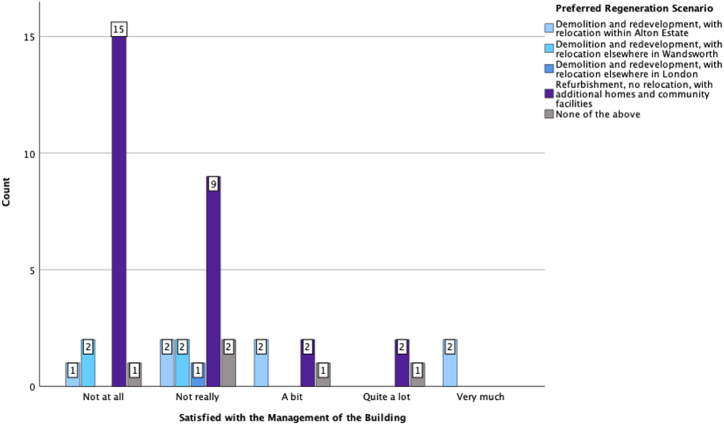
Grouped bar chart of regeneration preference compared to participants' satisfaction with the management of the building.

To find out associations between the participants' preferred regeneration scenario and other conditions and preferences, we considered the regeneration scenario as a dependent variable and conducted different statistical analyses using SPSS. Fig. 6, Fig. 7, Fig. 8 are a graphic summary of the relationship between the community's regeneration preference and whether the participants agreed with the statement that they were happy to be relocated (questioned as ‘*How do you feel about the following statement: I prefer to live in another home*’); whether they felt attached to their homes (questioned as *‘How do you feel about the following statement: I feel attached to my home’*); and whether they felt satisfied with their living conditions (questioned as *‘How do you feel about the following statement: I am satisfied with my living conditions*’). The horizontal axis describes the regeneration options, and the vertical axis highlights the number of participants who selected the regeneration option grouped by the degree of agreement with being relocated (Fig. 6), being attached to their home (Fig. 7), or being satisfied with their living conditions (Fig. 8). A cursory glance at the graphs reveals a significant relationship between disagreement with relocation and agreement with home attachment, and with refurbishment as the preferred option for regeneration (Fig. 6, Fig. 7). Fig. 8, Fig. 9, Fig. 10 show a particularly interesting pattern suggesting that regardless of the participants' dissatisfaction level with their living conditions and management and maintenance of the buildings, the refurbishment scenario was the most popular scenario.

**Fig. 11:**
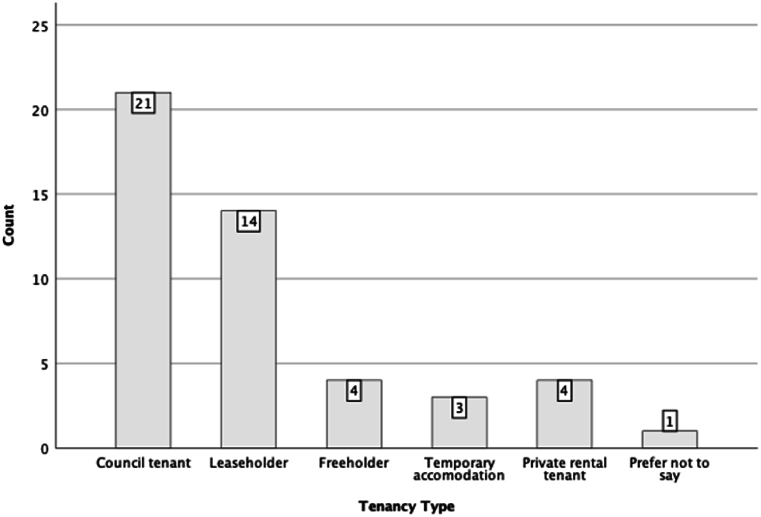
Histogram of residents' tenancy type by their counts.

**Fig. 12:**
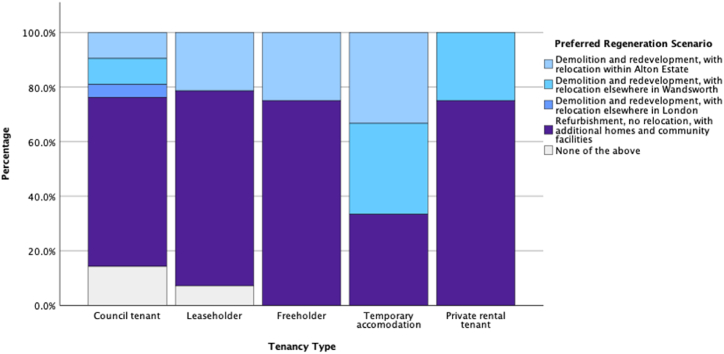
Stacked bar chart of participants' regeneration scenario scaled to values across different tenancy types.

**Fig. 13:**
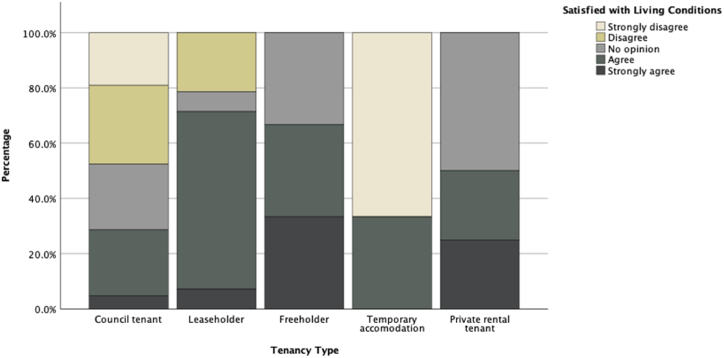
Stacked bar chart of participants' living satisfaction scaled to values across different tenancy types.

**Fig. 14:**
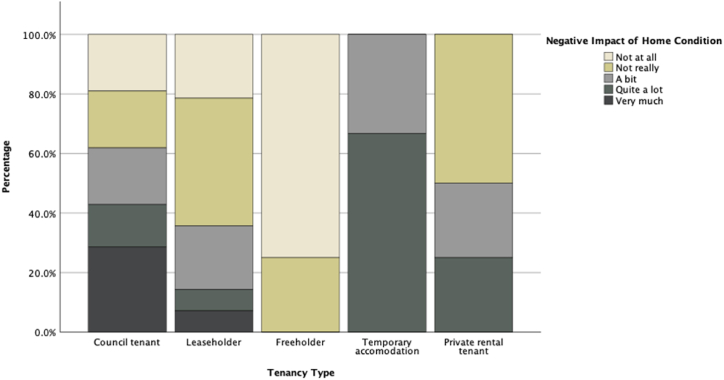
Stacked bar chart of participants' perception of the negative impacts of their home conditions scaled to values across different tenancy type.

To study other associations between participants' answers to the survey, we studied other key variables such as tenancy type, management satisfaction, and maintenance satisfaction as our dependent variables and compared them against other independent variables. Fig. 11, Fig. 12, Fig. 13, Fig. 14 illustrate potential relationships between the tenancy types and participants’ responses to the questions. In general, the council tenants and temporary accommodation residents appear to have lower satisfaction with their living conditions as opposed to the leaseholders, freeholders, and private rental tenants of the estate.

To understand the associations between the dependent and independent variables, crosstabulation and chi-square analyses have been used due to the nominal^7^ and ordinal^8^ scales of the studied data [65]. The results showed the assumption for chi-square tests for all the samples was not fulfilled, i.e. number of cells to be expecting counts less than 5 to be more than 20 %. Because of the violation of chi-square assumptions and the multiple categories of each variable (larger than 2 x 2 contingency), the Asymptotic Significance (2-sided) ρ-value under Likelihood Ratios has been studied for finding any 2-tailed associations between the dependent and independent variables, and Cramer's V for understanding the strength of associations between the variables [65,66]. Exploring the statistical significance of the results has been based on the acceptance of ρ-value being less than or equal to α = 0.10 at a confidence level of 90 % [71]. A benchmark for interpretation of the Cramer's V values [65] has been presented in Table 6. Testing the associations of the variables has shown some statistical significance (ρ-value ≤ 0.10) and a significant degree of association (Cramer's V ≥ 0.20) which have been illustrated in Table 7.

**Table 6:** An interpretation of Cramer's V values. Authors' reproduction [65].

Cramer's V Value	Interpretation of degree of association
0.0–0.10	Negligible
0.10–0.20	Weak
0.20–0.40	Moderate
0.40–0.60	Relatively strong
0.60–0.80	Strong
0.80–1.00	Very strong

**Table 7:** Comparing the Cramer's V values of regeneration preference with other variables with statistical significance and the interpretation of their degree of significance [65].

*Regeneration Preference association with:*	ρ-*value*	*Cramer's V Value*	*Interpretation*
Satisfaction with ventilation	*0.05*	*0.41*	Relatively strong association
Satisfaction with home size	*0.05*	*0.46*	Relatively strong association
Preference to move to another home	*0.01*	*0.41*	Relatively strong association
Impact of moving outside the estate	*0.10*	*0.31*	Moderate association
Satisfaction with location	*0.06*	*0.56*	Relatively strong association
Home attachment	*0.04*	*0.41*	Relatively strong association

A relatively strong association of *home attachment* with different dependent variables can be concluded from the tables. Other significant associations between *regeneration preference* and independent variables relate to satisfaction with different conditions such as location, home size and ventilation, and issues such as relocation. The analyses provide preliminary evidence that the community's priorities concerning regeneration are mostly related to social and management issues.

Overall, the statistical tests have provided a level of understanding of the relationships between different variables of the study. General dissatisfaction with the management and maintenance of the buildings and the estate is obtained from the descriptive analyses. The general picture emerging from the statistical analyses shows that many of the community priorities are of a socioeconomic nature and possibly due to the poor maintenance and management of estate, issues that may arise again in a redevelopment scenario if they are not addressed. Participants’ preference for refurbishment over demolition scenarios, despite their dissatisfaction with the conditions of the buildings, provides somehow convincing evidence in favour of this statement. These findings have assisted in the classification of indicators such as *Refurbishment (Regeneration Preference), Ventilation*, *home attachment,* and *Disruption Stress* (stress caused due to disruptions as implications of demolition). The findings also suggest the need for further in-depth exploration of these priorities through qualitative approaches to identify the full list of indicators and criteria for the LCSA of the scheme.

### 4.3 Thematic analysis of qualitative data

The iterative process of coding the qualitative data across different datasets has allowed focusing on the most relevant insights from the participants [68].

Coding has been done based on predetermined as well as spontaneous origins [68], which is related to the mixed inductive (mostly) and deductive approach of the study. The predetermined codes were identified from the gaps in the field to meet the specific objectives of the project [68].

Iterative open coding was conducted through a bottom-up approach without any pre-coding. After narrowing down the codes, and once the inherent key attributes emerged, it was compared to the rest of the codes for the potential emergence of themes.

The identified codes emerging from the TA have been categorised into relevant themes. This classification, where possible, has adhered to the categories identified through the literature review while introducing new categories based on the community's priorities extracted from the case study.

*Mental Health* as a separate category from *Physical Health,* and *Socioeconomic Values* are the main categories that have emerged through the TA in addition to the findings of the literature review.

The most important indicators for the participants related to stakeholder involvement in decision making, maintenance and management of the estate, refurbishment strategies, community and communal facilities, damp and mould, thermal comfort, disruption stress, energy savings, housing provision, and accessibility.

The findings of this reflexive TA and triangulation of the findings have assisted in identifying the community's most relevant indicators and criteria. Table 8 presents the list of identified codes interpreted as indicators and grouped to have the least overlapping of the themes, and avoiding duplication.

**Table 8:** Alphabetical List of the identified indicators and criteria.

Theme/Criterion	Code/Indicator
Accessibility	
	Provision of Inclusive Access, Access to all Amenities
Community Facilities and Amenities	
	Communal Facilities, Community Facilities, Greenery, Kids and Youth Facilities, Outdoor Spaces, Retail Amenities
Design Strategies and Innovation	
	Regeneration Plans, Design Aesthetics, Heritage, Building Functionality, Heritage, Spatial Program, Technological Matters,
Climate Change	
	Energy Saving, Embodied Carbon Emissions
Environmental Impacts	
	Environmental Impacts, Renewables, Air Pollution, Water Pollution
Local Ecological Impacts	
	Preservation of Trees, Biodiversity, Water Reuse
Whole Life Costs	
	Operational Costs, Social Rent
Management & After Care	
	Building Maintenance, Building Refurbishment, Building Management, Construction Management, Transparency, Waste Management
Material Strategies and Circulatory	
	Re-use of materials, Material Sources, Durability of Materials
Mental Health	
	Regeneration Anxiety, Disruption Stress, Archetype Anxiety, Relocation Stress, Uncertainty over Regeneration
Physical Health	
	Airtightness, Condensation, Damp and Mould, Thermal Comfort, Ventilation, Lighting
Safety and Security	
	Antisocial Behaviour, Fire Safety Concerns, Security
Social Values	
	Community Involvement, Residents' Satisfaction, Social Cohesion Hidden Impacts, Social Ties, Long-term Concerns
Socioeconomic Values	
	Fuel Poverty, Affordability, Housing Provision, Local Economic Revitalisation, Security of Tenure
Transport and Movement	
	Car Park, Green Means of Transport, Transport Networks

### 4.4 Stakeholder feedback

**Fig. 15:**
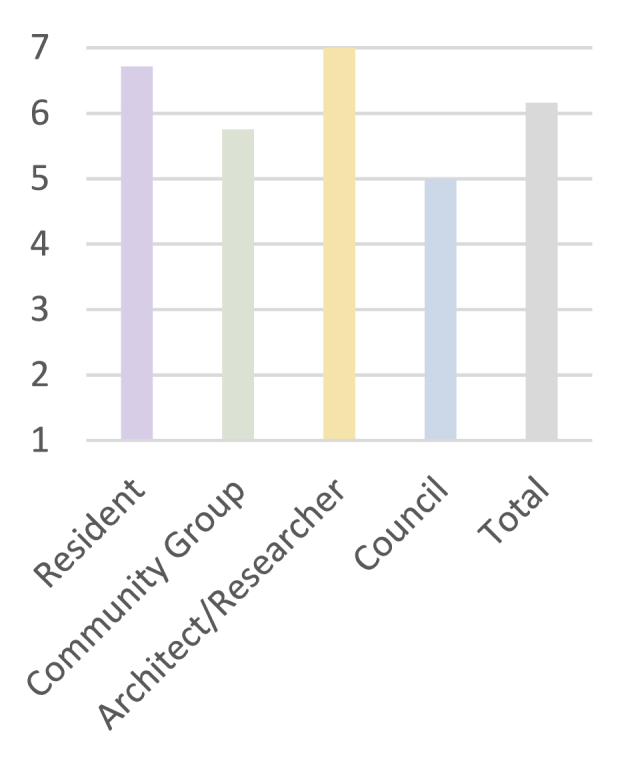
Mean evaluation values across different stakeholder groups.

The identified criteria, shown in Table 8, were presented to the different stakeholders of the estate for feedback at an in-person workshop. Results of the questionnaire on the participants’ agreement with the identified criteria illustrate the overall satisfaction of the twenty-one participants with the identified criteria. Fig. 15 presents these results with a total mean value of 6.16, and a standard deviation of 1.12.

## 5 Discussion

The empirical mixed methods case study has bridged the gaps identified through the review of literature.

- The incomprehensiveness of criteria has been addressed by identifying a relevant list of criteria that transparently responds to any sustainability indicator and is a pertinent representation of a spectrum of criteria for the scope of LCSA.
- Lack of community focus and engagement in identifying the sustainability indicators and criteria has been mitigated by in-depth community engagement and exploration of community priorities for recognising the sustainability indicators and criteria.
- Finally, the gap in lack of contextual considerations for sustainability criteria of estates has been bridged by focusing on a case study of a housing estate to identify a relevant list of indicators and criteria.

To respond to the research aim for identifying a set of criteria for a community-centred LCSA of estate regeneration schemes, the findings of the mixed methods case study have been triangulated with the results of the literature review. This has resulted in identifying a list of added indicators and introducing a new set of criteria for the scope of the LCSA of this case study and similar studies.

Some of the sustainability indicators that were identified through the analysis of the case study include Fuel Poverty, Security of Tenure, Housing Provision, Occupant Rate and Density, Overcrowding, Home Attachment, Civic Impacts of Regeneration Scenario, and Personal Impacts of Regeneration Scenario. Although some of these indicators have been noted in other research on Social Impact Assessment of housing estates in London [31,34], our proposed added criteria were not identified in these studies.

In introducing the list of criteria, considerations have been taken into account for the description of the criteria to be as much as possible non-technical. In the grouping of the identified indicators, avoiding double-counting at the level of criteria has been an important aspect of the proposal as it is one of the main assumptions of lifecycle-based methodologies [26,72]. A careful categorisation of indicators not only prevents double-counting of the indicators in different criteria, but it is more tangible to the stakeholders and can be used for goal and scope definition and communication of the results of the studies. Based on the findings of the literature review and empirical research, the criteria presented in Table 9 have been introduced for the scope of LCSA in the context of estate regeneration. This approach to the grouping of indicators facilitates data collection for global and local ranking of the proposed criteria. Given the changing nature of assessment criteria with the current needs of the market, the categorisation allows flexibility for the future identified indicators to be added under the proposed categories. The highlighted added criteria have been explained below.

**Table 9:** Authors' proposed list of criteria for LCSA of estate regeneration schemes.

	Criteria
1	Climate Change
2	Environmental Impacts & Strategies
3	Local Ecologic Impacts & Strategies
4	Material Strategies and Circularity
5	Whole Life Cost
6	Physical Health
7	Accessibility
8	Safety and Security
9	Transport & Movement
10	Community Facilities and Amenities
11	Social Values
12	Maintenance and Management
13	Design Strategies and Innovation
14	Mental Health
15	Socioeconomic Values

**Climate Change** – Due to the increased attention to Energy and Net-Zero buildings, and in response to the growing concerns over global warming [73] this category has been introduced separately from the rest of the global Environmental Impacts. This criterion includes indicators such as Operational Carbon Emissions, Embodied Carbon Emissions, Energy Savings, Energy Efficiency, and Cumulative Fossil Energy Consumption.

***Local Ecologic Impacts & Strategies*** – Ecologic values are included under “Management” category in some of the [29] schemes, and under “Impacts on Neighbourhood” category in the BS ISO 21931–1:2022 [26], while LEED V4.0 [27] has “Biodiversity” as one of its categories. Identifying Local Ecologic Impacts & Strategies as a separate criterion avoids double counting of this category, and includes relevant indicators such as biodiversity, ecologic enhancement, and water strategies.

***Socioeconomic Values*** – Whole Life Cost criterion includes all the associated costs within the building's life cycle. It has been separated from other economic values outside the system boundary. In practice, the economic impacts are in many cases assessed through Life Cycle Cost (LCC) while the *Socioeconomic Values* outside the system boundary are hardly considered in the economic evaluation of different regeneration scenarios. In the context of regeneration schemes of housing estates, the socioeconomic values (e.g., affordability, security of tenure, local revitalisation, and fuel poverty) are vital to the community as the main stakeholders, as the findings of the case study suggest.

***Mental Health*** – Mental health can be interpreted to be included in “health and wellbeing”, a criterion that has been noted in most assessment schemes. However, the indicators of those criteria are mostly related to physical health issues and do not specify issues related to mental health as sustainability indicators. BS ISO 21931–1:2022 [26] classification of “*Consideration of Different Stakeholders during the Planning/Design Phases*” under the “*Architectural Quality*” criterion is vague and does not clearly portray the importance of issues around mental health for the community in relation to disruption and estate regeneration. The significance of mental-health-related issues for the community in the context of housing estate regeneration has been an important finding of our quantitative and qualitative research. The focus of assessment frameworks on physical health when discussing health and wellbeing has led us to include *Mental Health* as a separate criterion. In our proposal, *Mental Health* criterion relates to issues such as civic and personal impacts of regeneration scenarios.

***Physical Health****– Health and Wellbeing criterion has been renamed* as *Physical Health* to cover matters such as indoor air quality, ventilation, thermal comfort, acoustic comfort, and visual comfort.

The findings of the qualitative analysis of this case study have somehow been consistent and complementary to the findings of the quantitative analysis and the literature review. These findings highlight the priorities of the communities that are not completely reflected in the criteria categorisation of current platforms for the sustainability assessment of the regeneration schemes, such as *Socioeconomic Values*, and *Mental Health and Wellbeing*. In pursuit of a profound LCSA scope with a justifiable indicator selection [30], the indicators have been interpreted from identifying the priorities of the community. Categorisation of the indicators has resulted in a global classification of criteria for the sustainability indicators.

Our findings suggest that the contextual nature of estate regeneration requires an in-depth study of each project to identify the indicators of the LCSA framework based on the priorities of the stakeholders of the scheme. The findings of the literature review have presented some inconsistency and subjectivity in the criteria of sustainability assessment schemes and frameworks (Table 2). While identified sustainability indicators are specific to the context of this case study, the transparent clarification and careful classification of the global sustainability criteria can be used for accessible communication with the stakeholders and interpretation of the results. This approach to having a comprehensive list of criteria enables equal distribution of attention to all identified criteria, shifting away from energy-centred or economically-driven assessments. The overall satisfaction of the estate stakeholders with the identified criteria supports the findings of this study.

## 6 Conclusion

This paper has proposed a community-centred scope for LCSA of an estate regeneration scheme in London. The proposal is through primary and secondary data collection and analysis. A case study with the community of a housing estate has been conducted which has contained surveys, an interview, and an evaluation questionnaire. Through the literature review, statistical analysis of quantitative data, thematic analysis of the qualitative data, and triangulation of the results of the study, the following conclusions have been drawn.

• Lack of a standardised set of preliminary sustainability indicators across different frameworks, not including the stakeholders and their priorities in identifying and classifying the sustainability indicators, and lack of contextual considerations, are some of the main gaps in literature that this study has covered in relation to identifying a scope for LCSA of regeneration schemes.
• The statistical analysis of the data provided evidential support that the dissatisfaction of the participants with their estate is mostly related to issues around poor maintenance and management of the estate. Participants' preference for a refurbishment scenario over demolition scenarios, despite their dissatisfaction with the conditions of the buildings, supports this theory.
• The results obtained from the qualitative research presented the importance of socioeconomic and mental health issues among the participants' most important priorities such as maintenance and management of the estate, community facilities, and issues related to physical health.
• The findings of TA and triangulation of the results of the case study and literature review have introduced a comprehensive list of criteria with new categories including *Mental Health and Wellbeing*, and *Socioeconomic Values.* Due to the global attention to achieving Net Zero carbon emissions, Climate Change has been separated as a separate criterion from other Environmental Impacts and Strategies. Local ecologic Impacts and Strategies have also been introduced as a separate criterion.
• The stakeholders' satisfaction with the identified criteria is consistent with the findings of this study for a relevant list of criteria for LCSA of estate regeneration schemes.
• The findings of this paper support the importance of engaging with the communities and exploring their priorities to identify a plausible scope for LCSA in the context of estate regeneration.
• The proposed set of criteria of this study helps in shifting away from energy or economically driven and biased assessments and provides a comprehensive and accessible approach for communication with the stakeholders and interpretation of the LCSA results.

Our dataset for the case study was limited to the study sample of the community of Alton Estate, therefore, our findings of sustainability indicators for the appraisal of Alton Estate are not generalisable beyond the study sample. However, while the proposed list of indicators elicited from the community is specific to the context of this study, the criteria categorisation of identified codes based on the literature review and supported by the case study can be used in similar studies to introduce a more relevant and descriptive list of criteria which avoids double counting of indicators. We would encourage researchers to examine these findings beyond the population studied in this case study, and in different contexts and locations. Moreover, although the elicitation of data from the stakeholders is context-based, the explored participatory methodology is context-free and can be applied to other settings in finding a stakeholder-centred LCSA scope. Future research can confirm the suitability of the proposed criteria by exploring the approaches of this research on other housing estates in different locations. We would also recommend exploring the priorities of other stakeholders (such as the client team, planning authorities, design team, sustainability specialist and academics, and construction team) to identify a multi-stakeholder LCSA scope in the context of estate regeneration.

## 7 Declarations

This research and the collaborative study [62] have followed the ethics’ guidance from University College London (UCL). The collaborative study was a knowledge exchange project and has been approved by the UCL Ethics Committee, Professor Michael Heinrich (Approval ID Number 9089/003). The rest of the research has obtained a Low Risk Ethics Approval from UCL, Bartlett School of Environment, Energy and Resources, Dr Francesco Aletta (Approval ID Number 20210907_IEDE_PGR_ETH). This study complies with General Data Protection Regulations (GDPR) and is registered under the UCL Data Protection (Registration Reference Number Z6364106/2021/06/235). The main survey and the co-design workshops have been conducted collaboratively with a team of UCLresearchers [62] – Pablo Sendra as the principal investigator, and Sahar Nava as one of the researchers – in partnership with Alton Action and Just Space, funded by the Knowledge Exchange Innovation fund, Higher Education Innovation Fund (10.13039/501100013589Research England), managed by UCLInnovation & Enterprise. Online written consent was obtained from the participants for the survey study and verbal informed consent was obtained for co-design workshops.

This research is funded by 10.13039/501100000266Engineering and Physical Sciences Research Council (10.13039/501100000266EPSRC) – Grant Reference EP /N509577/1 and EP /T517793/1.

We would like to thank all our participants from the Alton Estate community, and Alton Action group who have generously taken the time and energy to take part in our research.

## Data availability statement

Due to the confidentiality of the survey responses, the data associated with the survey of this study has not been deposited to a publicly available repository.

## CRediT authorship contribution statement

**Sahar Nava:** Writing – review & editing, Writing – original draft, Visualization, Methodology, Investigation, Formal analysis, Conceptualization. **Zaid Chalabi:** Writing – review & editing, Supervision. **Sarah Bell:** Writing – review & editing, Supervision. **Pablo Sendra:** Writing – review & editing. **Esfand Burman:** Writing – review & editing, Supervision.

## Declaration of competing interest

The authors declare the following financial interests/personal relationships which may be considered as potential competing interests: Sahar Nava reports financial support was provided by 10.13039/501100000266Engineering and Physical Sciences Research Council. Pablo Sendra reports financial support was provided by Knowledge Exchange Innovation fund, Higher Education Innovation Fund (10.13039/501100013589Research England). If there are other authors, they declare that they have no known competing financial interests or personal relationships that could have appeared to influence the work reported in this paper.

## Footnotes

1 Secondary or tertiary impacts are referred to as mid-point impacts, while primary impacts are characterised as end-point impacts.

2 Although intended for Non Domestic projects, the scale and description of many estate redevelopment projects would qualify them to be assessed under this scheme.

3 In September 2022, after a change of political party in Wandsworth council, the council decided to stop the masterplan and explore different approaches to regeneration.

4 Paul A. Harris, Robert Taylor, Robert Thielke, Jonathon Payne, Nathaniel Gonzalez, Jose G. Conde, Research electronic data capture (REDCap) - **A metadata-driven methodology and workflow process for providing translational research informatics support**, *J Biomed Inform. 2009 Apr;42(2):377-81*.

5 PA Harris, R Taylor, BL Minor, V Elliott, M Fernandez, L O'Neal, L McLeod, G Delacqua, F Delacqua, J Kirby, SN Duda, REDCap Consortium, **The REDCap consortium: Building an international community of software partners**, *J Biomed Inform. 2019 May 9 [*https://doi.org/10.1016/j.jbi.2019*.103208]*.

6 REDCap Citation, Available at https://projectredcap.org/resources/citations/(Accessed 25 January 2022).

7 Question about the participants' preferred regeneration scenario had the below choices to be selected as an answer: 1) Demolition and redevelopment, with relocation within Alton Estate; 2) Demolition and redevelopment, with relocation elsewhere in Wandsworth; 3) Demolition and redevelopment, with relocation elsewhere in London; 4) Refurbishment of existing homes, right to stay in your current home (no relocation), with additional homes, community facilities and shops built on the estate; 5) None of the above.

8 Many of the questions in the survey gave the choice of ordinal answers, such as: 1) Strongly disagree; 2) Disagree; 3) No opinion; 4) Agree; 5) Strongly Disagree.
